# The Binding of Concanavalin A to the Surface of Intact and Denuded Sea Urchin Eggs Affects the Fertilization Process by Altering the Structural Dynamics of Actin Filaments

**DOI:** 10.3390/cells14231867

**Published:** 2025-11-26

**Authors:** Nunzia Limatola, Marinella Pirozzi, Davide Caramiello, Jong Tai Chun, Luigia Santella

**Affiliations:** 1 Department of Research Infrastructures for Marine Biological Resources, Stazione Zoologica Anton Dohrn, 80121 Napoli, Italy; 2 Department of Biology and Evolution of Marine Organisms, Stazione Zoologica Anton Dohrn, 80121 Napoli, Italy; chun@szn.it; 3 Institute of Endotypes in Oncology, Metabolism, and Immunology “G. Salvatore,” National Council of Research (IEOMI-CNR), Via Pietro Castellino 111, 80131 Napoli, Italy; marinella.pirozzi@ieos.cnr.it; 4 Department of Marine Animal Conservation and Public Engagement, Stazione Zoologica Anton Dohrn, 80121 Napoli, Italy; davide.caramiello@szn.it

**Keywords:** acrosome reaction, sea urchin eggs, jelly coat, species-specificity recognition, bindin, vitelline layer, Concanavalin A, fertilization, actin, calcium

## Abstract

Sea urchin eggs are surrounded by a network of extracellular matrix, consisting of the jelly coat (JC) and vitelline layer (VL). While the voluminous JC evokes acrosomal reaction in the approaching sperm, the tight VL ensheathing the plasma membrane of the subjacent microvilli is known to be the subcellular site where ‘sperm receptors’ reside. In this study, we have examined the roles of JC and VL at fertilization in a combinatorial approach utilizing two different pretreatments of the eggs: (i) incubation with dithiothreitol (DTT) in alkaline seawater to remove JC and VL, (ii) masking the egg extracellular matrix with a carbohydrate-binding protein concanavalin A (Con A). Surprisingly, the results showed that the DTT-denuded eggs still engulfed sperm at fertilization, even more effectively than intact eggs, as multiple sperm entered. On the other hand, Con A appeared to interfere with sperm entry in a dose-dependent manner and to delay the onset of the Ca^2+^ wave in intact eggs after the cortical Ca^2+^ release, representing sperm–egg fusion. This prolonged time lag in triggering the Ca^2+^ wave at fertilization was associated with compromised dynamics of the subplasmalemmal actin filaments in Con A-pretreated eggs. By using Alexa Fluor 633 Con A and BPA-C8-Cy3, respectively, we also report unprecedented fluorescent labeling of the egg JC and the spontaneous ‘acrosomal protrusion’ on the head of *Paracentrotus lividus* sperm diluted in natural seawater. Combined with electron microscopy observations of intact and denuded eggs, our results suggest that the glycoconjugate on the egg surface contributes to the fertilization signal transduction, affecting the Ca^2+^ wave via actin cytoskeletal changes and sperm entry.

## 1. Introduction

For more than a hundred years, sea urchins have served as an excellent experimental model for studying fertilization in vitro because a large number of gametes can be easily manipulated and observed in the Petri dish containing seawater, i.e., in experimental conditions closely resembling what happens at sea. However, despite the enormous knowledge and insights gained from sea urchins, our understanding of fertilization in this marine organism remains incomplete and is a work in progress. The reports in the literature describe a generalized scheme of fertilization-associated reactions, including sperm activation and the acquisition of motility triggered by seawater, which increases intracellular pH and the respiration rate, as well as by egg-derived sperm-activating peptides that induce Ca^2+^-dependent sperm chemotaxis [1]. It has also been shown that the components of the egg jelly (jelly coat, JC) surrounding sea urchin eggs trigger the acrosome reaction (AR) in vitro, during which the acrosomal vesicle in the anterior portion of the sperm head undergoes exocytosis, which allows species-specific binding of the fertilizing sperm to the egg [2,3,4,5,6,7,8]. Since the JC fractions of some sea urchin species induced the AR in homologous sperm, it was suggested that the species-specific induction of the AR was due to the structural uniqueness of the JC in the pattern of sulfation and the glycosidic linkage of the polysaccharides [1,9]. The AR comprises extension of the acrosomal process (AP) via actin polymerization, which exposes bindin at the tip of the sperm head. This adhesive protein interacts with the sperm receptors on the thin vitelline layer (VL) of the egg, which tightly adheres to the microvilli (MVs) containing actin emerging from the egg membrane by “vitelline posts” [10,11,12,13]. If the VL on the egg surface is digested by proteolytic enzymes, the fertilizability of eggs is reduced as a result of disrupted sperm-binding sites on the VL [14]. Additional studies have shown that during the cortical reaction of a fertilized egg, specific proteases are released from secretory vesicles located beneath the egg membrane, known as cortical granules (CGs). These proteases are deposited into the perivitelline space (PS), resulting in the breakdown of the linkages between the VL and the plasma membrane. The action of proteases facilitates the separation of the VL from the egg plasma membrane and abolishes the sperm receptor sites, which contributes to the slow block to polyspermy [15,16,17]. In line with this idea, trypsin inhibitors, which block the elevation of the fertilization envelope (FE) and sperm detachment, induce polyspermy [16,18,19]. Structural proteins extruded from the CGs deposit in the PS, and subsequently, the elevating VL hardens to form the FE to protect the embryo [20,21,22].

Following fertilization, it was also possible to analyze the nature of the subcellular proteins released by sea urchin eggs following the exocytosis of the CGs at fertilization or after removal of VL by dithiothreitol (DTT) treatment [18,23]. The subsequent events of the fertilization process involving the penetration of the VL by the AP of the activated sperm and fusion between the membranes of sperm and egg have been poorly understood due to the rapidity of the process and to the fact that only one of the multiple sperm arriving on the egg surface will fuse with the egg plasma membrane [19,24,25,26].

According to the prevailing view, the exposed bindin on the sperm AP is essential for the sperm to recognize and bind to its receptors in the egg VL [27]. However, it has been shown that the fertilization response of *Paracentrotus lividus* eggs can occur even if the JC and VL are structurally altered or removed prior to insemination [26,28,29,30,31], suggesting that these layers may not be essential for sperm activation. In sea urchin eggs, the fertilization process is characterized by an initial phase involving the depolarization of the plasma membrane, which coincides with Ca^2+^ influx a few seconds after the fusion of the sperm and egg membranes [32,33,34]. The Ca^2+^ response induced by sperm is affected by the shape of the MVs, which contain actin filaments, as well as by the structural integrity of the CGs and vesicles associated with the egg’s plasma membrane. Preserving the structural integrity of the egg cortex is essential for the proper physiological changes that occur during egg activation [35,36,37,38]. The fertilization Ca^2+^ signals are concomitant with the depolymerization of F-actin in the outer region of the egg’s cytoplasm (ectoplasm), which initiates formation of a dimple at the sperm entry point and brings about the contraction of the egg and its separation from the VL [39,40,41,42].

This early Ca^2+^-dependent cortical reaction is a prerequisite for the subsequent metabolic activation and embryonic development. During this late fertilization phase (5 min after insemination), the egg exhibits the development of K^+^ conductance, MVs elongation, and cortical actin polymerization induced by an intracellular pH increase [19,43,44,45,46,47,48]. The latter change counteracts the depolymerization of F-actin on the egg surface, which occurs a few seconds after the addition of sperm [31,42]. The cortical actin remodeling at the site of sperm–egg binding involves the formation of a cone of actin filaments (fertilization cone, FC) to engulf the sperm, which was first visualized by the transmission electron microscope in sea urchin eggs inseminated in the presence of nicotine to induce polyspermy and thereby to increase the chances of containing multiple FC in the ultrathin sections [49,50]. It is important to note that recent studies have demonstrated that nicotine treatment of *P. lividus* eggs consistently induces polyspermic entry by significantly altering the dynamics of the egg’s F-actin, resulting in abnormal sperm penetration into the activated eggs [51].

Previous research has explored the interactions between fertilizing sperm and the surface of sea urchin eggs, with a particular focus on the role of glycoprotein components in sperm receptors. These studies demonstrated that the lectin Concanavalin A (Con A), which binds to various polysaccharides [52], can prevent fertilization in several sea urchin species at concentrations exceeding 0.1 mg/mL [53]. An investigation into the effect of Con A on sea urchin fertilization, involving the incubation of intact *Strongilocentrotus purpuratus* eggs with fluorescent Con A, revealed a strong affinity for binding to the VL sites but not to the JC sites. Additionally, the removal or alteration of the VL sites using dithiothreitol (DTT) [23] reduced the number of Con A binding sites on the plasma membrane of unfertilized eggs, indicating a low affinity [54]. Following fertilization of eggs deprived of the VL, the increased number of Con A binding sites on the egg plasma membrane was attributed to the insertion of the membranes of the CGs following their exocytosis [55]. These results indicated that the interaction of Con A with the “naked” plasma membrane of *S. purpuratus* eggs did not prevent the attachment and fusion of the sperm with the egg and the induction of the cortical reaction [54,55,56]. While the effect of Con A on several aspects of fertilization (e.g., gamete interaction, fertilization envelope formation, cleavage, etc.) is quite diverse depending on the sea urchin species, the reason for this difference has been poorly understood because the relationship between the Con A-binding carbohydrate fraction and the sperm receptor has not been well characterized [57]. The latter point calls for more stringent analyses of the timeline of gamete fusion, physiological responses (e.g., Ca^2+^ signaling and cytoskeletal changes), and sperm entry into sea urchin eggs pretreated with Con A.

In the present study, we exposed *P. lividus* eggs to various concentrations of Con A to examine the role of the VL glycoprotein in species-specific gamete interaction, and the morphological and functional aspects of the fertilization process. Upon insemination, the initiation of the sperm-induced Ca^2+^ signal representing the sperm–egg fusion has been used as a criterion with which to judge whether the binding of Con A with the VL carbohydrate residues could affect sperm–egg interaction and egg activation. Our electron microscopy examinations have revealed that 5 min of exposure of intact and denuded *P. lividus* to Con A significantly alters the topography of the egg surface, e.g., the ultrastructure of the VL, MVs, and CGs. In intact eggs, the changes in egg surface morphology induced by lectin incubation do not prevent sperm from interacting with and fusing to the egg’s plasma membrane. Additionally, these changes do not inhibit the initial sperm-induced Ca^2+^ signal at the egg’s periphery, known as the cortical flash (CF). However, they do significantly delay the onset of the subsequent Ca^2+^ wave (CW). Pretreatment with Con A inhibits the remodeling of the cortical actin cytoskeleton that typically occurs in fertilized eggs, thereby also guiding sperm entry. These characteristic effects of Con A on Ca^2+^ and actin cytoskeletal responses in the fertilized eggs were nearly nullified or alleviated in denuded eggs, suggesting that the carbohydrate moiety of the egg surface may play a role in transducing the fertilization signals. Another significant achievement in the present study is the unprecedented visualization of Con A binding to the outer JC, enabled by the use of a fluorescently labeled lectin in epifluorescence or confocal microscopy with a wider pinhole setting.

## 2. Materials and Methods

### 2.1. Gamete Preparation and Fertilization

Sea urchins (*P. lividus*) were collected in the Gulf of Naples from December to April and kept in a tank with circulating seawater at 16 °C. Spawning was induced by intracoelomic injection of 0.5 M KCl into female and male animals. Eggs were collected in natural seawater (NSW) filtered with a Millipore membrane (pore size 0.2 µm, Nalgene vacuum filtration system, Thermo Fisher Scientific, Rochester, NY, USA) and used for the experiments within 3 h after spawning. Denuded eggs were prepared by incubating intact, unfertilized eggs for 20 min in NSW containing 10 mM DTT (adjusted to pH 9.0 with NaOH) [26,30] to remove the extracellular matrix (the outer egg jelly and vitelline layer). For the insemination of the eggs, the dry sperm was diluted in NSW only a few minutes before fertilization at the final concentration of 1.84 × 10^6^ cells/mL.

### 2.2. Time-Lapse Movies of the Fertilization Process and Visualization of the Sperm in Intact and Denuded Eggs

The fertilization response (cortical structural and Ca^2+^ changes) of intact and denuded eggs with and without Concanavalin A (Con A) treatment was monitored by epifluorescence microscopy with a cooled CCD (charge-coupled device) camera (CoolSNAP HQ^2^ camera, Photometrics, Roper Scientific, Inc., Trenton, NJ, USA) mounted on a Zeiss Axiovert 200 inverted microscope (Carl Zeiss AG, Oberkochen, Germany) with a Plan-Neofluar 40×/0.75 and 20×/0.5 objectives and a CMOS camera (CoolSNAP Myo, Photometrics, Roper Scientific, Inc., Trenton, NJ, USA) mounted on a Zeiss Observer A1 with a Plan-Neofluar 20×/0.5 objective and an XBO 75 W lamp. To determine sperm entry, dry sperm were diluted afresh in NSW containing 5 µM of the nuclear fluorescent dye Hoechst-33342 (Sigma-Aldrich, St. Louis, MO, USA) for 30 s before fertilization. The male pronuclei inside the eggs were searched and counted 5 min after insemination by use of epifluorescence microscopy.

### 2.3. Scanning Electron Microscopy (SEM)

Intact or denuded *P. lividus* eggs were partitioned and preincubated in the presence or absence of Concanavalin A (Con A). Before and after insemination, aliquots of the eggs in each condition were fixed in NSW containing 0.5% glutaraldehyde (pH 8.1) for 1 h at room temperature, and then were post-fixed with 1% osmium tetroxide for another hour. After several rinses, the specimens were dehydrated in ethanol with increasing concentrations and then subjected to critical point drying using a LEICA EM CP300 (Leica Microsystems CMS GmbH, Wetzlar, Germany). The samples were coated with a thin layer of gold using a LEICA ACE200 sputter coater (Leica Microsystems CMS GmbH, Wetzlar, Germany) and were observed with a JEOL 6700F scanning electron microscope (Akishima, Tokyo, Japan).

### 2.4. Transmission Electron Microscopy (TEM)

For TEM analyses, intact or denuded eggs were treated in the same experimental conditions described for SEM observations. After fixation in NSW containing 0.5% glutaraldehyde (pH 8.1) for 1 h at room temperature they were post-fixed with 1% osmium tetroxide and 0.8% K_3_Fe(CN)_6_ for an additional hour at 4 °C. After washing three times in NSW, the samples were rinsed twice in distilled water for 10 min and subsequently treated with 0.15% tannic acid for 1 min at room temperature. After extensive rinsing in distilled water (10 min, three times), the samples were dehydrated in acetone of increasing concentration, transferred to a solution of acetone and SPURR and embedded in SPURR. Resin blocks were sectioned with a Leica EM UC7 ultramicrotome (Leica Microsystems CMS GmbH, Wetzlar, Germany), and the ultrathin sections (70 nm in thickness) were observed with FEI TECNAI G2 Spirit BioTWIN 120 kV Transmission Electron Microscope (FEI Company, Eindhoven, The Netherlands) equipped with a Veleta CCD digital camera (Olympus Soft Imaging Solutions GmbH, Münster, Germany).

### 2.5. Chemicals, Reagents, and Recombinant Proteins

Concanavalin A (Con A) and the fluorescent Con A (Alexa Fluor 633) were purchased from Sigma Aldrich and Invitrogen (Molecular Probes, Inc., Eugene, OR, USA) and dissolved in distilled water and 0.1 M Sodium bicarbonate, respectively. Unless specified otherwise, all reagents used in this study were purchased from Sigma-Aldrich. The recombinant protein LifeAct-GFP [58] was bacterially expressed [30,31,39] from a plasmid kindly provided by Dr. A. McDougall of Sorbonne University, France. A fluorescently labeled version of the branched synthetic polyamine N, N, N’, N’ [tetrakis(aminopropyl)octamethylenediamine] (abbreviated to BPA-C8) has been used as a molecular probe with which to visualize the extracellular matrix of starfish and sea urchin gametes [29,59,60]. BPA-C8-Cy3 utilized in the present study was generously provided by Dr. J.-M. Lehn and his colleagues, following a detailed chemical procedure described in a previous publication [29]. Live *P. lividus* sperm and eggs were diluted in NSW containing 50 µM BPA-C8-Cy3, and observed under a Leica TCS SP8X confocal laser-scanning microscope equipped with a white light laser and hybrid detectors (Leica Microsystems, Wetzlar, Germany).

### 2.6. Microinjection, Ca^2+^ Imaging, Fluorescence, and Confocal Microscopy

Freshly spawned intact eggs were microinjected using an air-pressure transjector (Eppendorf FemtoJet, Hamburg, Germany), as previously described [29,34]. To detect the sperm-induced intracellular Ca^2+^ increase, 500 µM Calcium Green 488 conjugated with 10 kDa dextran was mixed with 35 µM Rhodamine Red (Molecular Probes, Eugene, OR, USA) in the injection buffer (10 mM Hepes, 0.1 M potassium aspartate, pH 7.0) and microinjected into the eggs before insemination. The relative fluorescence signals of the cytosolic Ca^2+^ were acquired with a cooled CCD camera (CoolSNAP HQ2, Photometrics, Roper Scientific, Inc., Trenton, NJ, USA) mounted on a Zeiss Axiovert 200 microscope with a Plan-Neofluar 20×/0.5 objective to simultaneously monitor the sperm-induced Ca^2+^ signals in several eggs (average of *n* = 6) and a CMOS camera (CoolSNAP Myo, Photometrics, Roper Scientific, Inc., Trenton, NJ, USA) mounted on a Zeiss AXIO Observer A1 with a Plan-Neofluar 20×/0.5 objective at about 3 s intervals. The data were analyzed with MetaMorph (Universal Imaging Corporation, Molecular Devices, LLC, San Jose, CA, USA). Following the formula F_rel_ = [F − F_0_]/F_0_, where F represents the average fluorescence level of the entire egg, and F_0_ is the baseline fluorescence, the overall Ca^2+^ signals were quantified for each moment. F_rel_ was expressed as RFU (relative fluorescence unit) for plotting the Ca^2+^ trajectories.

### 2.7. Visualization of Actin Filaments and Lectin Binding

LifeAct-GFP (10 µg/µL, pipette concentration) or Alexa Fluor 568 phalloidin (10 µM, pipette concentration) was microinjected into live intact or denuded eggs to visualize the cortical F-actin. To assess the increased thickness of the egg cortical actin cytoskeletal layer at fertilization, *P. lividus* eggs were microinjected with LifeAct-GFP. This fluorescent probe specifically binds to F-actin and G-actin [58] with a strong preference to the actin pool near the egg plasma membrane [42]. The eggs were then inseminated, and the average height of the LifeAct-GFP signals at the cortex was measured from the same eggs before and 5 min after fertilization using the formula h = A/C, where A represents the total area of the signals within the subplasmalemmal layer. C denotes the circumference of the eggs (calculated as radius × 3.14) based on the pixel size in the images analyzed using MetaMorph. To visualize the binding of Con A, intact or denuded eggs were exposed to 200 µM Alexa Fluor 633 Con A in NSW. Before and after fertilization, intact and denuded eggs treated with Alexa Fluor 633 Con A were observed using a Leica TCS SP8X confocal laser scanning microscope equipped with a white light laser and hybrid detectors (Leica Microsystems, Wetzlar, Germany), with different pinholes as described in Section 3.

### 2.8. Statistical Analysis

The numerical MetaMorph data were compiled and analyzed with Excel (Microsoft Office 2010) and reported as mean ± standard deviation in all cases in this manuscript. Paired *t*-test and One-way ANOVA were performed with Prism 5.0 (GraphPad Software), and *p* < 0.05 was considered statistically significant. Results with *p* < 0.05 indicated statistical significance of the difference between the two groups, assessed using Tukey’s post hoc tests.

## 3. Results

### 3.1. Effect of Concanavalin A Exposure on the Fertilization Envelope Elevation and Sperm Entry

Unfertilized *P. lividus* eggs were treated with different concentrations of Concanavalin A (Con A) in seawater for 5 min and subsequently inseminated in the presence or absence (after washing) of the lectin (see below, Table 1). To monitor the progress of fertilization, the elevation of the fertilization envelope (FE) was examined, and the number of sperm incorporated into eggs was counted using the vital fluorescent DNA dye Hoechst 33342.

Con A treatment at a concentration of 1 mg/mL for 5 min completely inhibited the formation of the FE, which could not be reversed by washing off Con A with fresh NSW before insemination, in line with the previous reports for *P. lividus* and other sea urchin species [53,57].

While the 5 min pretreatment with 1 mg/mL Con A consistently prevented fertilization and sperm entry in *P*. *lividus* eggs, the same pretreatment with a lower dose of Con A (200 µg/mL) displayed a more subtle effect. A noticeable feature of these eggs pretreated with this dose of Con A is a certain anomaly at the initial phase of fertilization. In many sea urchin species, the beginning phase of the FE elevation is marked by a momentary invagination of the cytoplasm, which forms a prominent concave (here defined as ‘dimple’) on the egg surface at the sperm entry site [39,40,41].

**Figure 1:**
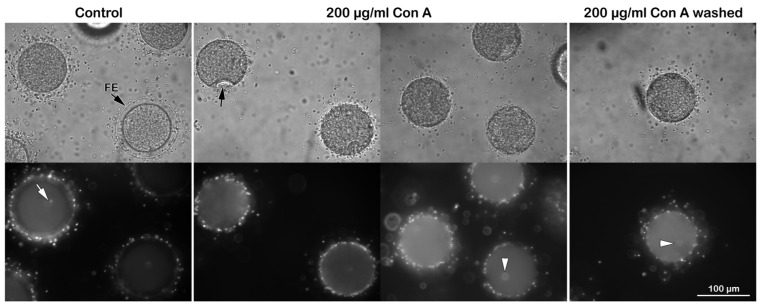
Effect of Concanavalin A (Con A) on fertilization. Intact *P. lividus* eggs were pretreated with 200 μg/mL Con A for 5 min before insemination. The bright-field image obtained 5 min after fertilization shows the elevation of the fertilization envelope (FE) in the control, but this elevation is blocked in eggs preincubated with Con A. In these eggs, a noticeable dimple (black arrow) on the surface remained visible even 5 min after insemination and showed no sign of the FE elevation. The representative epifluorescence images (lower row) show the fertilizing sperm stained with Hoechst-33342 inside the control eggs 5 min post-insemination (white arrow). The white arrowhead points to a female pronucleus.

In the eggs of *P. lividus*, in their best physiological conditions, a dimple forms approximately 10 to 15 s after fertilization. This temporary phenomenon has been observed in fixed samples [25,26,29,42], but it is often overlooked when examining the fertilization process with a light microscope. The live imaging of intact eggs at fertilization illustrates the sequential events during dimple formation (see Video S1 and its corresponding captioned time frame). This event coincides with the separation of the vitelline layer (VL) from the egg membrane after the occurrence of the intracellular Ca^2+^ wave (CW). At the same time, cortical actin is rearranged and cortical granules (CGs) are exocytosed, leading to the formation and elevation of the fertilization envelope (FE). Then, the egg readily resumes its spherical shape as the FE lifts over the entire egg surface within 1 min, as shown in the control egg (Figure 1 bright-field image, upper panel).

The intact eggs exposed to Con A before insemination exhibited a lower fertilization rate, as judged by the average number of sperm present within each egg. Although 40 out of 60 examined eggs were penetrated by one sperm, the remaining 20 inseminated eggs contained no sperm at all (Table 1). The epifluorescence microscopic images (low panels in Figure 1) show a single sperm (white arrow) that has fused with the female pronucleus. By contrast, in the eggs preincubated with 200 μg/mL Con A (5 min) before fertilization, the dimple persisted for over 5 min after fertilization (Figure 1, black arrow in the bright-field image). Still, the fertilization process apparently did not proceed in most cases. The FE did not elevate, and washing out Con A before insemination made no difference in reversing the result (Figure 1). Since the transient appearance and disappearance of the dimple beneath the separated VL reflects contraction of the cortical cytoskeletal elements [41], our result raises the possibility that the dynamics of the actin cytoskeleton might have changed in intact eggs pretreated with 200 μg/mL Con A.

**Figure 2:**
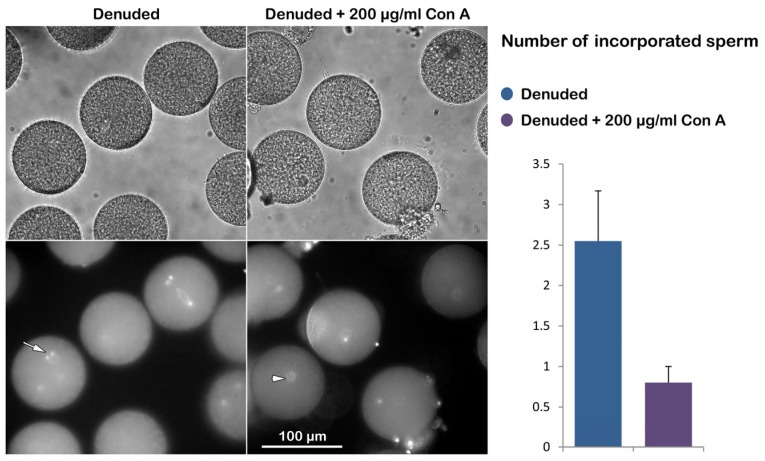
Effect of Con A on fertilization of denuded *P. lividus* eggs. The bright-field image in the upper panel shows that the FE did not elevate as the VL had been removed before insemination. The characteristic dimple did not form on the egg surface, although multiple sperm entered the egg. See the numerous sperm stained with Hoechst-33342 inside the denuded eggs 5 min post-insemination (white arrow), distinct from the female pronucleus (white arrowhead) in denuded eggs viewed by epifluorescence microscopy. To avoid cluttering the images, only the representative sperm and female pronucleus were marked with an arrow and arrowhead, respectively. The histograms on the right show the average number of sperm per egg for each experimental condition. Data on denuded eggs, with or without Con A pretreatment, were obtained from 2–3 different animals, yielding 60 or 40 eggs, as shown in Table 1.

Our previous studies have demonstrated that removing the JC and VL promotes polyspermic fertilization [26,30]. This finding indicates that the interaction and fusion of multiple sperm with the egg surface occur at the plasma membrane rather than at the VL [61]. We then examined the effect of Con A on the fertilization response of the denuded eggs. Thus, *P. lividus* eggs were treated with 10 mM of DTT at pH 9 for 20 min to remove the associated VL and JC. The absence of the JC is already evident, as the two neighboring denuded eggs come into contact due to the lack of extracellular layers that would otherwise repel them (Figure 2, upper panel, light microscopy image). After insemination, 60 of 60 denuded eggs were fertilized by multiple sperm (Figure 2, white arrow in the epifluorescence image), resulting in a 100% polyspermic fertilization rate. However, when these denuded eggs (n = 60) were pretreated with 200 µg/mL Con A prior to insemination, the polyspermy rate was significantly alleviated (Table 1). Specifically, 48 eggs were fertilized by only one sperm, while the remaining 12 eggs exhibited no sperm entry at all (Figure 2, right column).

### 3.2. Visualization of Con A Binding on the Egg Surface

We then used Alexa Fluor 633 Concanavalin A (Alexa Fluor 633 Con A) to visualize the surface receptors on denuded eggs (Figure S1). The fluorescent lectin signals were localized only on the VL of intact eggs when the confocal microscope was set to a pinhole of a diameter of 1.0 Airy Units (AU). This confocal setup effectively blocks out-of-focus light during image formation. At this setting, it is only possible to detect the fluorescence of the lectin on the VL tightly attached to the egg plasma membrane and not the peripheral JC (n = 12). However, examination of the samples under the confocal microscope using a pinhole aperture (hole) at a larger size (4 AU instead of 1), i.e., non-confocal modality, allowed us to obtain brighter Alexa Fluor 633 Con A images representing the JC associated with the VL. Indeed, the Fluor Con A labeling of the JC and VL (n = 12, intact eggs) was also detected by the CCD camera utilizing epifluorescence microscopy to capture the sperm-induced Ca^2+^ imaging (Figure S2). It is noteworthy that the Alexa Fluor 633 Con A also labels the plasma membrane of unfertilized eggs (n = 15) from which the VL and JC have been removed (Figures S1 and S2B). Due to the absence of the VL, this weaker fluorescence is attributed to the low-affinity binding of the lectin to the egg plasma membrane, which has been reported in the previous studies on other sea urchin species [54]. Alternatively, it cannot be ruled out that the faint Alexa Fluor 633 Con A signals on the denuded egg might be contributed by some remnant of VL that intermittently survived the DTT treatment.

### 3.3. Effect of Concanavalin A Exposure on the Surface of Intact Sea Urchin Eggs Before and After Fertilization, Visualized with Electron Microscopy (Scanning and Transmission)

#### 3.3.1. Topography and Ultrastructure of Intact *P. lividus* Eggs Before and After Fertilization

**Figure 3:**
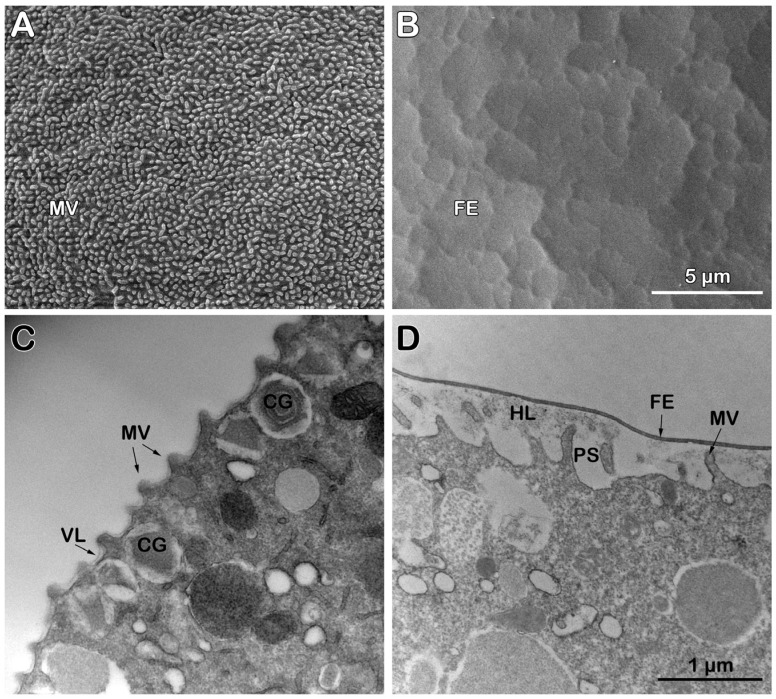
Scanning and transmission electron micrographs of the intact *P. lividus* eggs before and after fertilization. (**A**) A scanning electron micrograph shows microvilli (MVs) regularly distributed on the surface of an unfertilized egg. (**B**) Five minutes post-insemination, FE forms and elevates over the surface of the activated egg. The scale bar in panel (**B**) also applies to (**A**). (**C**) A transmission electron micrograph reveals the surface of an unfertilized egg with MVs covered by the vitelline layer (VL), along with secretory cortical granules (CGs) located beneath the egg’s plasma membrane. (**D**) Five minutes after insemination, elongated MVs amid the hyaline layer (HL) fill the perivitelline space (PS) beneath the fertilization envelope (FE). The scale bar in panel (**D**) also applies to (**C**).

Examination of the surface topography of intact, unfertilized *P. lividus* eggs using scanning electron microscopy (SEM) has confirmed our previously published results, which indicate that the JC adhering to the VL is lost during the fixation process—a well-documented phenomenon in the literature [25,26]. Owing to that, the SEM image of the fixed *P. lividus* eggs exhibited regularly spaced microvilli (MVs) protruding from the egg surface and tightly covered by the indistinguishable VL (Figure 3A). After fertilization, VL detaches itself from the egg plasma membrane to form the fertilization envelope (FE) (Figure 3B), which is aided by exocytosis of the cortical granules (CGs) at the egg plasma membrane. Indeed, transmission electron microscopy (TEM) revealed that the thin VL covers the egg plasma membrane MVs; CGs with various luminal morphologies are also evident (Figure 3C). Five minutes after insemination, CGs became scarce due to the exocytosis of their content into the perivitelline space (PS). This cortical fertilization reaction lasts approximately 1 min and contributes to the elevation of the FE over the entire egg surface (Figure 3D). Concurrently, the hyaline layer (HL), which aggregates following CGs exocytosis, is visible between the MVs elongated in the perivitelline space (PS). The separated VL from the egg surface during the cortical reaction is now transformed into a ticker FE (Figure 3D).

#### 3.3.2. Effect of Con A Preincubation on the Topography and Ultrastructure of Intact Eggs

**Figure 4:**
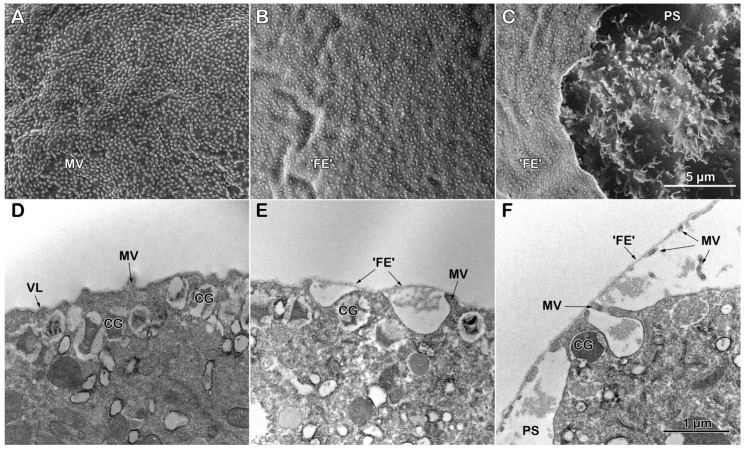
Scanning and transmission electron microscopy micrographs showing the fertilization response of intact *P. lividus* eggs pretreated with Con A before insemination. (**A**) Microvilli (MVs) on the surface of the unfertilized eggs after 5 min incubation with Con A. (**B**,**C**) Con A-pretreated egg 5 min after insemination, which shows the nascent and inapt fertilization envelope (‘FE’) covering the zygote surface. (**C**) In the perivitelline space (PS) of the fertilized eggs, MVs elongation is observed, where the ruptured ‘FE’ reveals the MVs in the PS. Panels (**A**–**C**) are on the same scale. (**D**) An intact unfertilized egg preincubated with 200 μg/mL Con A (5 min), showing secretory cortical granules (CGs) located deeper within the egg’s cytoplasm below MVs. (**E**,**F**) Con A-pretreated eggs 5 min after fertilization; the induction and propagation of CGs exocytosis are inhibited. Only isolated events of CGs exocytosis occurred, resulting in ‘FE’ formation. (**F**) Bound to Con A, the ‘FE’ of the intact eggs may have failed to detach from the MVs 5 min after insemination. Note that the ‘FE’ is thinner than usual due to the incomplete exocytosis of the CGs. The scale bar in panel (**F**) also applies to (**D**,**E**).

The binding of the lectin on the surface is expected to alter the egg’s structural dynamics at fertilization. Indeed, brief incubation (5 min) of the intact eggs with 200 µg/mL Con A readily induced a slight undulation of the egg surface, as shown in the SEM (Figure 4A). This waviness makes MVs in the transmission electron microscopy (TEM) less distinguishable from the baseline of the egg plasma membrane (Figure 4D). During fertilization, the modified surface of the egg becomes more pronounced when viewed under scanning electron microscopy, as shown in Figure 4B. In contrast to the control depicted in Figure 3B, the nascent fertilization envelope (‘FE’) does not detach from the surface of intact eggs that were exposed to Con A before insemination. This is demonstrated by the observation that MVs tips remain anchored to the inner side of the VL, which is the precursor to the FE. Consistent with the morphological changes observed in the cortical reaction, only certain intermittent areas exhibited signs of CGs exocytosis (‘FE’) and MVs extension when examined using TEM, as depicted in Figure 4E,F. Even there, the incomplete ‘FE’ appears thinner than usual. Where there is a localized elevation of the ‘FE’, its fortuitous rupture visualizes the MVs in the perivitelline space (PS) (Figure 4C). Still, the MVs elongation is rather inhomogeneous and irregular, making it difficult for the ‘FE’ to detach from MVs tips (Figure 4F). The impeded separation of the VL from the egg plasma membrane, which is necessary for the formation of the FE, is consistent with findings reported in other species of sea urchins. This effect is caused by structural alterations induced by Con A treatment [57].

#### 3.3.3. Topography and Ultrastructure of Denuded Eggs Before and After Fertilization

**Figure 5:**
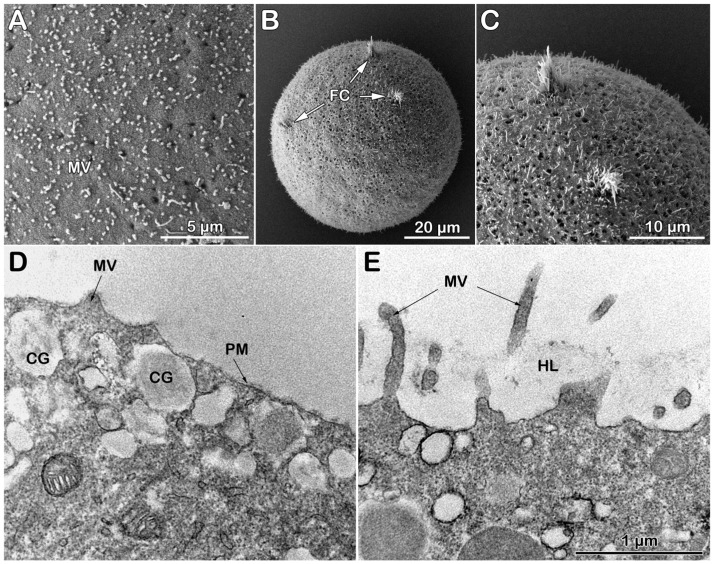
Scanning and transmission electron micrographs of the denuded *P. lividus* eggs before and 5 min after fertilization. (**A**) Topography of a denuded egg before fertilization. Note that there are fewer microvilli (MVs), and they appear altered. (**B**) Inseminating denuded eggs results in polyspermic fertilization, as evidenced by the formation of three visible fertilization cones (FC, arrows) incorporating sperm. (**C**) Magnified image of the fertilization cones in panel (**B**). Note that, albeit scarce, MVs are still elongated from the surface of the fertilized egg amid numerous holes. The FE is absent because the VL had been removed before insemination. (**D**) A denuded egg with the altered appearance of MVs and cortical granules (CGs). (**E**) By 5 min post-insemination, the CGs exocytosis took place as judged by the absence of the CGs from the visual plane and by the presence of the hyaline layer (HL) formed by the extruded contents of CGs. Despite the MVs elongation, no visible FE was formed. The scale bar in panel (**E**) also applies to (**D**).

As previously reported [26,30], the removal of the VL, which tightly adheres to the MVs, drastically alters the MVs morphology and reduces their number on the egg surface. Indeed, the SEM micrograph of the *P. lividus* eggs pretreated with DTT at pH 9.0 to remove the VL already shows morphological changes on the surface of the denuded egg (Figure 5A). After insemination (5 min), the consistent tendency of polyspermic fertilization displayed by the denuded eggs (Table 1) is also reflected in the multiple (three) fertilization cones (FC) observed in the SEM image of the egg (see Figure 5B, labeled FC). Despite the numerous sperm entries, it is noteworthy that the denuded eggs at fertilization fail to raise the FE because its precursor VL had been removed by the DTT treatment [26,30]. A closer examination of the fertilization cones in the SEM micrograph revealed non-uniform MVs elongation at the site of sperm entry (Figure 5C), probably due to the reorganization of the F-actin within and around the MVs, as was previously reported [29]. Interestingly, MVs on the fertilized egg surface are interspersed among numerous holes, presumably through which the CGs have released their contents after fusing with the egg plasma membrane directly into the seawater [16,17,18,19]. The TEM of a denuded egg before fertilization (Figure 5D) confirms the SEM observations of a reduced number of MVs on the egg surface and the lack of the continuous line representing VL in the intact eggs (compare it with Figure 3C). TEM images also confirm that the separation of the VL from the egg plasma membrane at fertilization and its subsequent thickening to form the FE are precluded by the DTT treatment (Figure 3D and Figure 5E for comparison). Although non-uniformly, MVs elongation on the fertilized egg surface still occurs in areas where they are present. The hyaline layer (HL) made from CGs exocytosis was retained during the fixation amid the elongated MVs in the absence of the FE elevation (Figure 5E).

#### 3.3.4. Morphological Effect of Con A on the Denuded Eggs Before and After Fertilization

**Figure 6:**
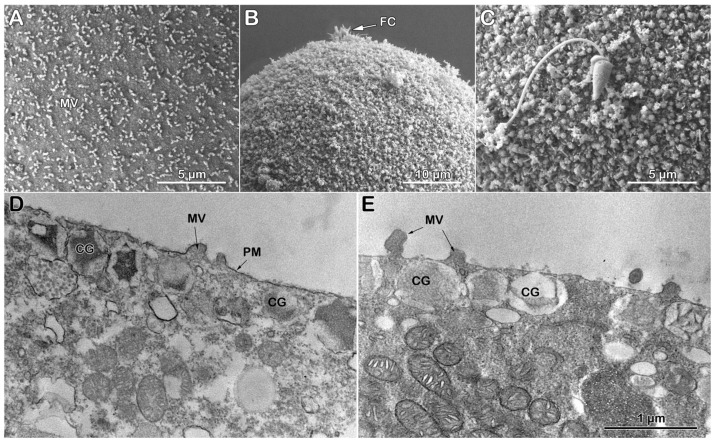
Effect of Con A on the fertilization of the denuded eggs of *P. lividus*. (**A**) A denuded egg exposed to Con A shows a reduced number of microvilli (MVs) and alterations in surface morphology. (**B**) When Con-A-treated denuded eggs are inseminated, the resulting fertilization cones (FC) exhibit structural modifications. (**C**) A higher magnification image of the surface of a fertilized egg reveals a significant lack of MVs elongation compared to the image shown in Figure 5C. (**D**) Before fertilization. A denuded egg treated with Con A displays the altered morphology of MVs, cortical granules (CGs), and cytoplasmic ultrastructure. (**E**) After fertilization (5 min), the presence of CGs beneath the plasma membrane is still evident on the surface of the activated egg. Additionally, MVs are consistently flattened. Note the absence of FE elevation due to removing the vitelline layer from the unfertilized egg. The scale bar in panel (**E**) also applies to (**D**).

More dramatic effects of Con A on denuded eggs become particularly evident after fertilization. The SEM micrograph in Figure 6A shows the surface of an unfertilized denuded egg treated with Con A (200 µg/mL) for 5 min, which appears similar to that of untreated denuded eggs (see Figure 5A for comparison). In contrast, the denuded eggs pretreated with Con A exhibited changes in the structure of the fertilization cone (FC). After sperm penetration, its outward profile appeared flatter, and there was almost complete inhibition of MVs elongation (see Figure 6B,C). This suggests that Con A pretreatment led to significant alterations in the structural plasticity of the MVs on the egg surface. The TEM micrograph in Figure 6D illustrates the reduced number of MVs on the surface of a denuded egg with CGs located beneath the egg plasma membrane. After exposure to Con A, the ultrastructure of the denuded eggs exhibits more cortical modifications, as judged by the altered morphology of CGs and vesicles. At fertilization, rather than elongating, as seen in untreated denuded eggs (see Figure 5E), many MVs collapsed, forming enlarged spherical tips (Figure 6E). Such changes in the MVs have been shown to result from depolymerization of the actin filaments that form the MVs [62].

### 3.4. Alteration of the Cortical F-actin in Intact and Denuded Eggs in Response to Con A

#### 3.4.1. Effect of Con A on Intact Eggs at Fertilization

The exposure of *P. lividus* eggs to Con A led to changes in the ultrastructural organization of the egg surface and cortex. Furthermore, preincubation of the intact eggs with 200 μg/mL Con A significantly inhibited sperm entry at fertilization, a process known to be heavily dependent upon the actin filaments of the egg’s surface [26,29,34]. Thus, to investigate the relationship between Con A treatment and actin cytoskeletal changes at the plasma membrane, we visualized the actin filaments by microinjecting the eggs with bacterially expressed LifeAct-GFP and incubating them in NSW containing the membrane dye FM1-43.

**Figure 7:**
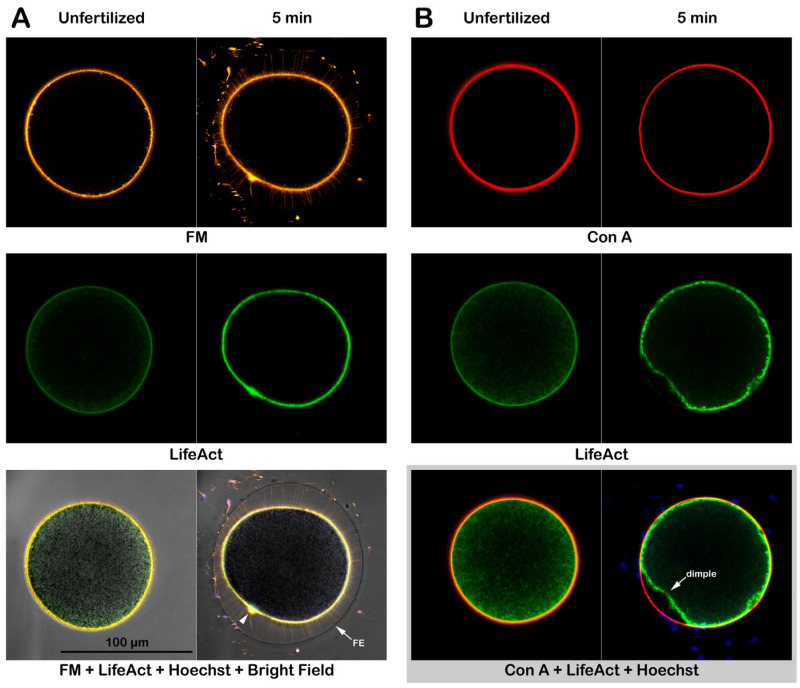
Cortical reaction and F-actin changes in intact eggs at fertilization. To visualize the changes in the plasma membrane, F-actin, and the extracellular matrix during fertilization, intact *P. lividus* eggs microinjected with LifeAct-GFP (green) were inseminated in the presence of the corresponding fluorescent markers: FM1-43 (orange color), Alexa-Con A (red), and the sperm were stained with the DNA dye Hoechst 33342. (**A**) Confocal microscopic images of the control eggs without Con A preincubation before and after (5 min) insemination. (**B**) Intact eggs with Con A preincubation (200 μg/mL, 5 min) before and after insemination. Note: (i) in the left merged fluorescent and bright field view, the sperm appear yellow instead of blue because of the presence of FM1-43 in the media, (ii) arrowhead refers to the fertilization cone engulfing the sperm with its tail still in the perivitelline space. (iii) Con A preincubation inhibited the propagation and elevation of the fertilization envelope (FE).

By 5 min after fertilization of intact eggs, the formation of F-actin spikes (MVs extension) in the perivitelline space (PS) was evidenced by FM1-43, which clearly delineated the plasma membrane ensheathing the extending MVs below the fully elevated fertilization envelope (FE) (Figure 7A). The MVs, especially their basal part, are also labeled by LifeAct-GFP [30]. Still, the signal is usually weaker because the free space within MVs is often too tight for the bulky protein probe to diffuse. Hence, LifeAct-GFP primarily visualized the thickening of the actin cytoskeletal layer in the cortical region of the zygote (Figure 7A, middle panel) 5 min after insemination. Indeed, the average height of the circumferential layer of the cortical actin cytoskeleton visualized by LifeAct-GTP was more than twofold increased from 0.86 ± 0.34 μm (eggs before fertilization) to 1.97 ± 0.21 μm (same eggs 5 min after fertilization), as summarized in Table 2 (n = 6, *p* < 0.001). When these intact eggs were preincubated with 200 µg/mL Con A for 5 min before insemination, the cortical reaction was subtly different (Figure 7B). The simultaneous visualization of the VL, stained red with Alexa Fluor 633 Con A, alongside LifeAct-GFP (green signal), revealed a persistent dimple on the zygote’s surface (bottom right image in Figure 7B). In the control eggs at fertilization, the cortical dimple readily disappears as the FE is elevated (Video S1), but in the eggs fertilized after the 5-minute preincubation with Con A, this dimple remained visible even 5 min after insemination, which is attributed to a significant delay in the contraction of eggs pretreated with the lectin (Video S2 and Figure 1). Moreover, it was found that the preincubation of intact eggs with Con A largely inhibits the post-fertilization thickening of the subplasmalemmal actin layer. The average thickness of the subplasmalemmal layer decorated by LifeAct-GFP signals after fertilization (1.20 ± 0.48 µm, n = 6) was not significantly higher than that of the same Con A-pretreated eggs before fertilization (0.88 ± 0.17 µm, n = 6, *p* = 0.240) (Table 2). Notably, at the site of the dimple, the cortical actin layer highlighted by LifeAct-GFP is recessed from the egg surface delineated by the red signal of Alexa Fluor 633 Con A (see Figure 7B for the overlay image at the bottom). This observation suggests that, in the presence of Con A, the intact eggs encounter difficulties in lifting the VL during fertilization, and that the dynamic function of the subplasmalemmal actin filaments may be compromised due to prior exposure to Con A.

#### 3.4.2. Effect of Con A on Denuded Eggs at Fertilization

**Figure 8:**
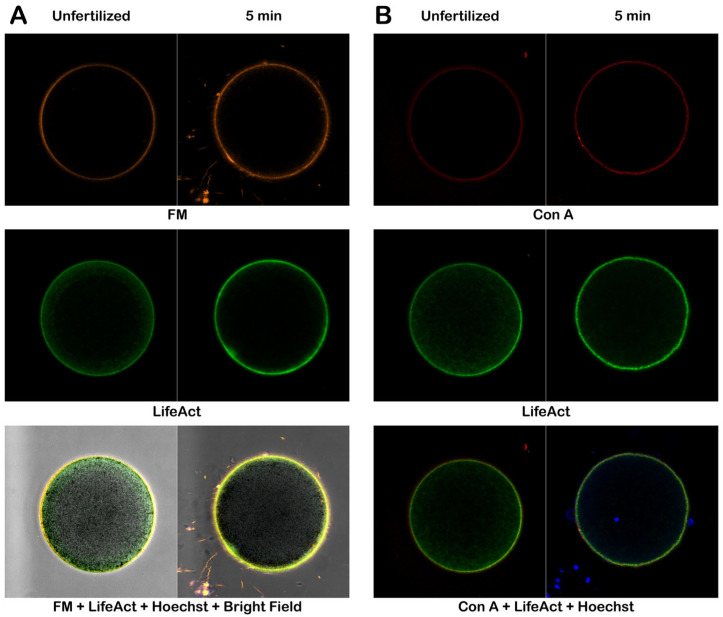
Effect of Con A pretreatment on denuded eggs at fertilization. Denuded eggs were incubated with or without 200 μg/mL Con A (5 min) before insemination. To visualize the plasma membrane, F-actin, and the extracellular matrix, the same eggs were microinjected or incubated with the corresponding fluorescent markers, as described in the Materials and Methods: FM1-43 (orange), LifeAct-GFP (green), and Alexa Fluor 633 Con A (red). (**A**) Confocal microscopic images of the denuded eggs without Con A pretreatment before and after fertilization. (**B**) Denuded eggs fertilized after 5 min incubation with 200 μg/mL. Blue signals represent sperm stained with Hoechst 33342.

The same experimental method used to visualize F-actin, the plasma membrane, and Con A binding was applied to the denuded eggs during fertilization (Figure 8). The confocal images of these fluorescent markers following fertilization were not as conspicuously altered as those of the intact eggs in the same condition. The thickness of the subplasmalemmal actin cytoskeletal layer of denuded eggs (0.76 ± 0.22 µm, n = 9) was only modestly increased after fertilization (1.16 ± 0.44 µm, n = 9, *p* < 0.05), as opposed to intact eggs that manifested more than twofold increase (Table 2). This scarce increase in the subplasmalemmal actin cytoskeletal layer in denuded eggs at fertilization was not much influenced by Con A pretreatment, as its average thickness 5 min after fertilization (1.55 ± 0.19 µm, n = 8) was still significantly greater than it was before insemination (1.24 ± 0.20 µm, n = 8, *p* < 0.01) (Table 2). In line with this marginal alteration in the cortical actin cytoskeletal layer, eggs that were denuded and pre-incubated with Con A at the time of fertilization did not exhibit appreciable signs of compromised surface egg contraction, a phenomenon already inhibited in untreated denuded eggs (refer to Video S3), because the Con A binding sites on VL are mostly removed (see Video S4).

#### 3.4.3. Effect of Con A on the Actin Filaments of Intact Eggs Probed by Alexa Phalloidin

**Figure 9:**
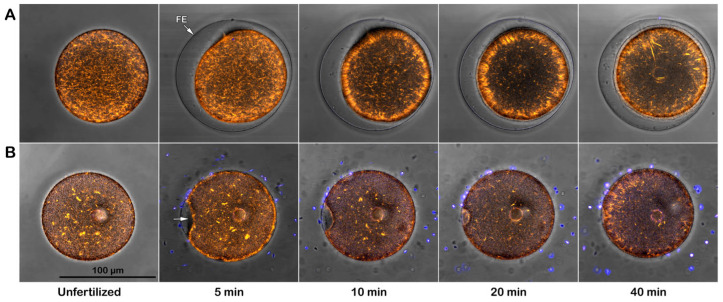
Effect of Con A on the actin filaments of intact eggs at fertilization probed by Alexa Phalloidin. Intact *P. lividus* eggs microinjected with Alexa Fluor 568-phalloidin were preincubated with or without 200 μg/mL Con A for 5 min and subjected to insemination. (**A**) The control egg’s fertilization response exhibits an elevation of the fertilization envelope (FE) and a progressive reorganization of actin filaments at the cortex. (**B**) Preincubation of the eggs with Con A altered the distribution of the actin filaments and inhibited their rearrangement at the cortex following fertilization. Note that the dimple (arrow) lingered on these eggs for 5 to 20 min amid failed FE elevation, implying decreased egg surface contractility caused by Con A.

While the elevation of the fertilization envelope (FE) is a prominent morphological event in a fertilized sea urchin egg, actin undergoes polymerization at the cortex and seemingly centripetal translocation [63]. In *P. lividus*, the cortical actin reorganization is discernible by 10 min after insemination, which can be most easily visualized by microinjecting 10 µM (pipette concentration) of Alexa Fluor 568 phalloidin into the cytoplasm of unfertilized eggs (Figure 9A). Since the cytosol further dilutes the probe within the egg, the used concentration of Alexa-phalloidin does not interfere with the physiological changes in cortical F-actin following fertilization of sea urchin eggs, as previously demonstrated [26,30,31,51,64]. When intact eggs (n = 12) were exposed to 200 μg/mL Con A before insemination, not only did the VL fail to separate from the egg surface, but the contractility of the egg cortex appeared to have changed. As a result, a modest dimple (Figure 9B, arrow) was formed and remained for 5 min (unlike the control egg) and lingered on the egg surface as long as 20 min. Furthermore, Con A pretreatment inhibited the actin polymerization and rearrangement observed in the cortex of the intact eggs at fertilization without Con A exposure. These findings suggest that Con A binding to the egg extracellular matrix may have interfered with a specific signal transducing mechanism by which the sperm triggers the reorganization of actin filaments in the fertilized egg.

#### 3.4.4. Fluorescent Labeling of Live *P. lividus* Gametes with Probes for Extracellular Matrix

**Figure 10:**
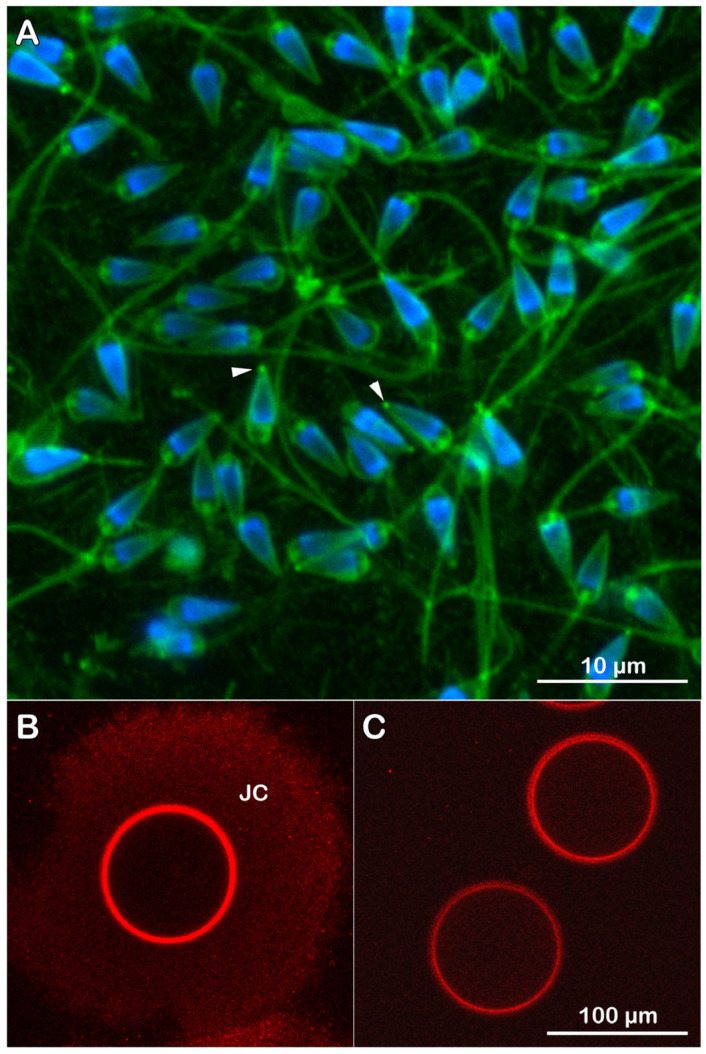
Fluorescent labeling of live *P. lividus* sperm and egg envelopes. (**A**) Fresh sperm were diluted in natural seawater (NSW) containing polyamine BPA-C8-Cy3 (green) and Hoechst-33342 (blue) and viewed with confocal microscopy. Note the presence of membrane protrusions on the sperm head, which is reminiscent of an acrosomal process (indicated by arrowheads). (**B**) An intact egg was labeled with Alexa Fluor 633 Con A and viewed with a confocal microscope in a non-confocal mode, allowing visualization of the jelly coat (JC). (**C**) Two denuded eggs labeled with Alexa Fluor 633 Con A and viewed in non-confocal mode.

It is widely accepted that the extracellular matrix surrounding the egg plays a critical role in initiating the sperm acrosome reaction (AR), a prerequisite for species-specific sperm–egg recognition and binding during fertilization. In other words, JC and VL are supposed to be required for sperm activation. Our previous study on the sperm-induced Ca^2+^ response in *P. lividus* eggs has demonstrated that sperm diluted in natural seawater (NSW) can fertilize the eggs even when the structural integrity of the VL is compromised or when the extracellular layers are removed [26,29,30]. Here, to test if some sea urchin sperm diluted with NSW in the absence of eggs already bear morphological differences, we fluorescently labeled the extracellular matrix of live *P. lividus* sperm with BPA-C8-Cy3 [29], and the eggs with Alexa Fluor 633 Con A (Figure 10). The results revealed the presence of a structural protrusion (Figure 10A, arrowheads) resembling an acrosomal process on the tip of some sperm heads. Although such a spontaneous ‘acrosomal protrusion’ was much shorter than that of the acrosomal processes on the sperm head of other species induced by the JC in vitro or at high pH and visualized with light and electron microscopy [19,65,66,67,68], its length was quite similar to that of the sperm observed by SEM on the surface of *P. lividus* eggs fertilized under physiological conditions [69]. These results appear to be in line with the previous report that a small percentage (ranging from 3 to 8%) of the sea urchin sperm in a given population already bear ‘ready-made AR’ [70]. On the other hand, the fluorescent labeling of intact *P. lividus* eggs with Alexa Fluor 633 Con A revealed the JC when the eggs were viewed in non-confocal mode (Figure 10B), as described in Section 2 (see also Figure S1). Although the egg extracellular matrix was removed (Figure 10C), the denuded eggs were still able to be fertilized by sperm diluted solely in NSW, without the aid of JC. These observations on the presence of an acrosomal-like structure in the sperm population diluted in JC-free NSW and their ability to fertilize denuded eggs raise a question about the indispensability of egg jelly-induced AR for fertilization in sea urchins.

### 3.5. Con A Binding to the Egg Surface Affects Sperm-Induced Ca^2+^ Signals in Eggs

#### 3.5.1. Ca^2+^ Responses in Intact Eggs Pretreated with Con A Before Insemination

**Figure 11:**
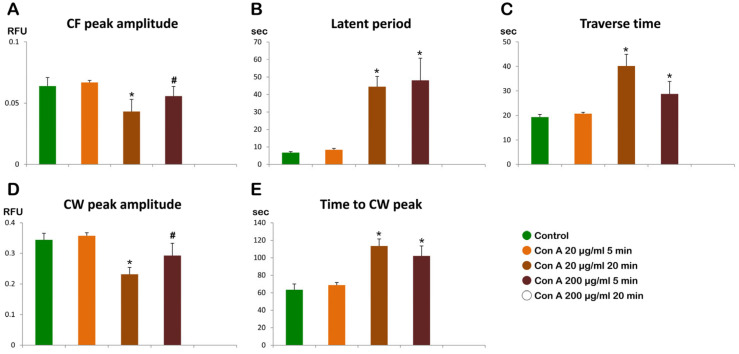
Histograms summarizing the Ca^2+^ responses in the control (intact, untreated) and Con A-pretreated eggs at fertilization. Each graph represents a specific aspect of the Ca^2+^ signals in the fertilized egg: (**A**) CF peak amplitude; (**B**) Latent period; (**C**) Traverse time; (**D**) CW peak amplitude; (**E**) Time to CW peak Note that the eggs pretreated with 200 µg/mL Con A (20 min) have no Ca^2+^ response. * *p* < 0.01, ^#^ *p* < 0.05.

The significant changes in the morphology of MVs and CGs observed in *P. lividus* eggs exposed to Con A, as visualized with electron microscopy, raised questions about how these lectin effects might interfere with sperm-induced Ca^2+^ signals, which are influenced by the structural alterations in the egg surface and cortex [26,31,34,38]. Therefore, we monitored the sperm-induced Ca^2+^ signals in intact and denuded eggs exposed to Con A before insemination. A typical Ca^2+^ response in fertilized eggs of the sea urchin displays several characteristic phases, as illustrated in Figure S3. A few seconds after the addition of sperm, a simultaneous and transient influx of Ca^2+^ at the cortex of the entire egg surface (cortical flash, CF) occurs, the amplitude of which is influenced by the length and morphology of the MVs [26,31,38]. Then, a Ca^2+^ wave (CW) starts at the sperm fusion site and propagates to the other side of the egg. The interval between the CF and the onset of CW is defined as ‘the latent period’, and the time required for the CW to arrive from the initiation site to the antipode is called ‘traverse time’, which is indicative of the speed of the Ca^2+^ wave. As the CW propagates, the intracellular Ca^2+^ level rises to a peak before it begins to decline. The duration needed for the intracellular Ca^2+^ level to reach its peak is referred to as the ‘time to peak’. Together with the maximal amplitudes of the CF and CW, these parameters could serve as criteria for characterizing changes in Ca^2+^ responses in fertilized eggs under various experimental conditions (Figure S3). Our experiments with intact eggs after preincubation with various doses of Con A in the media (5 min) revealed some notable alterations in the pattern of Ca^2+^ trajectories after fertilization (Figure S4 and histograms in Figure 11). In intact eggs fertilized without the Con A pretreatment (green lines in Figure S4A), the average Ca^2+^ responses at fertilization were characterized by: the CF of 0.064 ± 0.007 relative fluorescence unit (RFU) in amplitude (n = 10 eggs), latent period 6.7 ± 0.6 s (n = 10 eggs), peak amplitude of CW 0.34 ± 0.02 RFU (n = 10 eggs), and the traverse time about 20 s. The time-lapse movie shown in Supplementary Video S1 was made from simultaneous CCD recordings of the fertilization Ca^2+^ response and structural cortical changes in a representative egg. The intracellular Ca^2+^ release is likely to trigger the cortical contraction of the egg, which then contributes to the formation of the dimple-like structure that facilitates the separation of the VL from the egg plasma membrane [39,40,42]. This separation progresses across the entire egg surface over about 1 min, triggering CGs exocytosis and thereby forming a thick FE.

The examination of the intact eggs preincubated with Con A revealed that, regardless of the lectin concentration being used (1 µg to 1 mg/mL for 5 min), sperm physiology remained unaffected, as indicated by the unchanged time lag required by the sperm to reach the egg and trigger the CF. However, aside from this parameter, the graphs and histograms presented in Figure S4 and Figure 11 showed significant differences in the patterns of Ca^2+^ signals made by a 5 min preincubation with 200 µg/mL of Con A, whether or not Con A was removed from NSW before the insemination. The hallmark of the Ca^2+^ response in the intact eggs fertilized after Con A pretreatment was that there was a remarkably delayed initiation of the CW after the occurrence of the CF (Figure S4 and Figure 11B). Indeed, the latent period in these eggs was remarkably prolonged to 48.1 ± 12.6 s (n = 15) in comparison with the control eggs not pretreated with Con A (6.7 ± 0.6 s, n = 10, *p* < 0.01). Accordingly, this led to delayed and altered separation of the VL from the egg surface (Video S2).

Likewise, the exposure of unfertilized intact eggs to 200 µg/mL Con A before fertilization also influences the timing when the CW reached its peak intensity, which was measured at 101.9 ± 11.5 s (n = 15), as opposed to 63.3 ± 6.7 s (n = 10, *p* < 0.01, Figure 11E) of the control. On the other hand, the amplitude of the CW was significantly lowered, measuring 0.29 ± 0.04 RFU, compared to the control group (0.34 ± 0.02 RFU, n = 10, *p* < 0.05) (Figure 11D). In addition, the propagation of the CW was significantly slower, as judged by the traverse time: eggs pretreated with 200 μg/mL Con A (28.7 ± 5.1 s, n = 15), control eggs (19.3 ± 1.1 s, n = 10, *p* < 0.05) (Figure 11C and Figure S4). These subtle differences, revealed by the various parameters of the Ca^2+^ response, suggest that the externally administered Con A affects the cytoplasmic event, such as an increase in intracellular Ca^2+^.

The effect of Con A on the intracellular Ca^2+^ signaling in fertilized eggs is also dependent upon its dose and duration of incubation. When *P. lividus* eggs were preincubated with a 10-fold lower concentration of Con A (20 µg/mL) for 5 min, the sperm-induced Ca^2+^ signals were not significantly altered compared with the control eggs (Figure S4 and Figure 11). However, when the preincubation with the same dose of Con A (20 µg/mL) was prolonged 4 times more (20 min), the fertilization Ca^2+^ response was significantly altered (Figure S4), mimicking that of the eggs preincubated with a higher dose of Con A for a short time; the latent period became longer, and the CW amplitude lower (Figure 11).

#### 3.5.2. Ca^2+^ Responses in Denuded Eggs Pretreated with Con A Before Insemination

**Figure 12:**
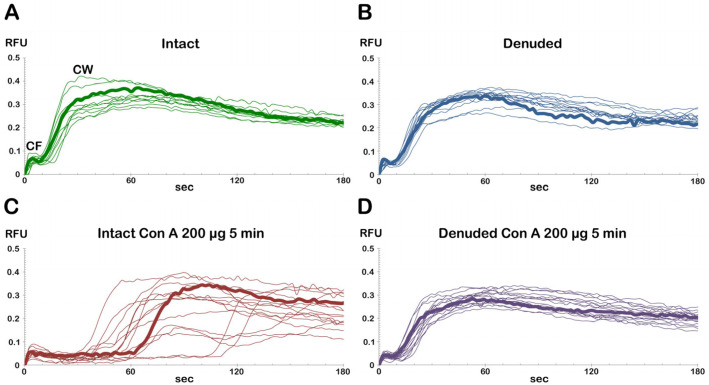
Comparisons of Con A effects on the fertilization Ca^2+^ responses in intact and denuded eggs. (**A**) Ca^2+^ signals of the intact *P. lividus* eggs fertilized without Con A preincubation. (**B**) Ca^2+^ responses of denuded eggs fertilized without Con A preincubation. (**C**) Ca^2+^ signals of the intact eggs inseminated after 5 min preincubation with 200 μg/mL Con A. (**D**) Ca^2+^ responses of denuded eggs fertilized after 5 min incubation with 200 μg/mL Con A preincubation. The moment of the first detectable cortical Ca^2+^ response (CF) was set as t = 0. Note that the Con A pretreatment significantly prolongs the latent period, i.e., the interval between the CF and the onset of CW in the intact eggs at fertilization, but the effect is lost in the denuded eggs. The bold line trajectories represent the Ca^2+^ responses visualized in the Supplementary Videos.

The denuded eggs were preincubated with or without 200 μg/mL Con A for 5 min, and their Ca^2+^ responses at fertilization were monitored and compared to those of the intact eggs (Figure 12). Despite the absence of VL and JC, the denuded eggs, which were washed multiple times in NSW at a pH of 8.1 to eliminate DTT, were successfully fertilized by sperm. These eggs exhibited a normal Ca^2+^ wave (see Figure 12B, blue lines) that was comparable to the wave observed in intact eggs (see Figure 12A). However, the average CF amplitude was noticeably reduced: 0.052 ± 0.003 RFU (n = 13, denuded eggs) as opposed to 0.064 ± 0.007 RFU for the intact eggs (n = 10 eggs, *p* < 0.05). This result makes sense because the CF is a Ca^2+^ event occurring at the egg surface. Other than that, most other aspects of the CW in denuded eggs were virtually the same as those of the intact eggs: (i) the average latent period for the denuded eggs was 5.7 ± 0.7 s (n = 13), and in intact eggs 6.7 ± 0.6 s; (ii) average CW amplitude of the denuded eggs (0.33 ± 0.01 RFU, n = 13) was practically the same as that of the intact eggs (0.34 ± 0.02 RFU, n = 10); (iii) the traverse time of the denuded eggs (19.3 ± 1.1 s, n = 10) was again not significantly different from that of the intact eggs (20.3 ± 1.5 s, n = 13).

Denuded eggs fertilized with or without Con A pretreatment (200 μg/mL, 5 min) made only minimal differences in the general patterns of the sperm-induced Ca^2+^ signals (compare Figure 12B,D). However, comparisons of the parameters characterizing the Ca^2+^ trajectories indicated that the amplitudes of both CF and CW were significantly reduced in the denuded eggs pretreated with Con A prior to insemination. While CF without Con A reached (0.052 ± 0.003 RFU, n = 13), the Con A pretreatment lowered the CF amplitude to 0.044 ± 0.005 RFU (n = 16, *p* < 0.01). Likewise, the amplitude of the CW was reduced from 0.33 ± 0.01 RFU (without Con A, n = 13) to 0.27 ± 0.03 RFU (n = 16 eggs, *p* < 0.01) as a result of Con A pretreatment (see also the Supplementary Video S4). More importantly, Con A pretreatment in the denuded eggs did not bring about the prolongation of the latent period between the CF and CW, which is consistently observed in the intact eggs fertilized after Con A pretreatment (compare Figure 12C,D). This finding suggests that the egg VL is involved in generating the CW following sperm–egg fusion.

## 4. Discussion

Fertilization of sea urchin eggs has been extensively studied across numerous species for over a century, resulting in a vast body of literature. Key findings indicate that fertilization is regulated by three significant interactions between the sperm and the egg. First, when the sperm contacts the outer jelly coat (JC) of the egg, the sperm acrosome reaction (AR) is triggered, and the sperm head extends an acrosomal process (AP) filled with F-actin and studded with the adhesive protein bindin. Second, bindin on the AP attaches in a species-specific manner to the glycoprotein of the vitelline layer (VL), which tightly covers the MVs of the egg membrane [71,72]. Finally, the fusion of the sperm with the egg membrane triggers the fertilization response, which is transduced into changes in the egg membrane potential and Ca^2+^ signals, leading to the exocytosis of the cortical granules (CGs) and eventually the formation of an elevated fertilization envelope (FE) by crosslinking and hardening the extruded CGs contents [56]. Thus, upon insemination of sea urchin eggs, both the egg JC and VL are supposed to play vital roles in fertilization. Furthermore, it has been postulated that early changes in the egg membrane potential (depolarization) at the moment of the gamete fusion serve as an electrically mediated fast mechanism to block the attachment of supernumerary sperm, thereby preventing polyspermy at fertilization [73].

Our recent findings, however, have challenged the prevailing view on how sperm fertilize sea urchin eggs by demonstrating the following key points. First, sperm can activate *P. lividus* eggs without undergoing the JC-induced AR. Second, species-specific recognition between gametes may occur at the egg plasma membrane rather than at the VL. These results are evident because denuded eggs, which lack both the VL and the JC, do not have difficulty attracting and binding sperm. Indeed, denuded eggs can undergo polyspermic entry, following multiple sperm bindings and fusion [26,30], Figure 2 and Figure 5. Ultimately, the fast block to polyspermy may be mediated through structural changes [74] rather than electrical ones [75,76]. This structural response involves the reorganization of actin filaments on the egg’s surface, enabling only a single sperm to successfully activate the egg [25,42,51].

In addition to being the location of the sperm receptor’s extracellular domain, which recognizes the bindin exposed on the AP of the sperm, the VL is also thought to play a crucial role in preventing multiple sperm entry (polyspermy). At fertilization, during the release of CGs content in the perivitelline space, proteases modify the structure of the VL, causing the bound sperm to detach. The subsequent complete swelling of the VL over the entire zygote surface, which leads to the formation of the FE, mechanically prevents any further interactions with additional sperm [77].

Nonetheless, this study reaffirms our previous findings that the VL of unfertilized eggs may obscure areas on the egg plasma membrane where multiple sperm can attach if its structural integrity is compromised [26,29,31]. Our findings, which highlight the significance of the morpho-functionality of the egg surface, align with earlier observations from sea urchin fertilization studies, suggesting that optimal physiological conditions for the gametes are crucial [74]. These studies claimed that sea urchin eggs “…….*if in best conditions are never polyspermic* ……… *Normally monospermic eggs can be rendered polyspermic by experimental treatment…..”* [78], which was corroborated in later studies [26,29,31,34]. Thus, the glycoprotein structure attached to MVs may aid in the fusion of only the fertilizing sperm with the egg membrane at a specific location that is not obscured by the VL (Figure 9 in [26]).

Research has shown that concanavalin A (Con A) inhibits various surface modifications on fertilized sea urchin eggs, including the formation of the FE, when the eggs are inseminated after exposure to the lectin. This inhibition has been interpreted as a blockage of fertilization and subsequent cleavage [14,53,54,57].

In this study, we investigated the effects of Con A on fertilization of *P. lividus* eggs to define the role of glycoprotein components of the VL in sperm–egg recognition and binding. Using fluorescent Con A, we visualized lectin binding to the glycoconjugates on the surface of intact and denuded eggs from which the VL had been stripped off. In addition to confirming Con A binding sites on the VL of intact eggs [54,55], we demonstrated, for the first time, its binding to the JC. Bound to the intact eggs, Con A inhibited the separation of VL across the entire egg surface after fertilization, leading to the failure of FE elevation and a dose-dependent inhibition in sperm entry.

This contribution shows that fertilizing sperm can still trigger the cortical reaction, albeit in an altered form, in intact eggs treated with Con A. One looming implication of our findings is that an event at the glycoconjugate in the extracellular domain, such as Con A binding, can affect intracellular signaling. The findings suggest that alterations in the extracellular matrix induced by Con A binding affect the structure and dynamics of F-actin on the egg’s surface. These morphological changes subsequently influence several early and late physiological events of the cortical reaction in the fertilized egg, which depend on the precise regulation of the actin cytoskeleton. Among these events are changes in microvilli morphology and CGs exocytosis, both of which are crucial for generating and propagating Ca^2+^ signals. One of the most intriguing findings from our study is that Con A pretreatment significantly prolongs the time to the onset of the Ca^2+^ wave during fertilization of intact eggs. However, this effect is not observed in denuded eggs (see Figure 12). This observation underscores the critical role of the VL in initiating the Ca^2+^ wave (CW). Since the VL is tightly anchored to the microvilli (MVs) on the egg surface, any changes to the VL can influence the dynamics of the cortical actin cytoskeleton. Our previous studies support this idea, showing that the latent period of the Ca^2+^ response in fertilized eggs is strongly correlated with the state of the egg’s cortical actin. Specifically, a delayed onset of the CW following the cortical Ca^2+^ release (cortical flash, CF) is consistently observed in *P. lividus* eggs that have been inseminated after treatment with actin-disrupting drugs such as cytochalasin B and latrunculin A [34]. Analogously, this latent period regarding the two Ca^2+^ events (CF and CW) is also reflected by electrophysiological measurements reported in previous studies. In these eggs, two electrical events occur across the egg plasma membrane in response to interaction with the fertilizing sperm. The first event is a step-like depolarization a few seconds after insemination, which corresponds to the CF in Ca^2+^ imaging. Then follows a fertilization potential that mirrors the CW in the fertilized egg. Interestingly, the time interval between the two electrical events is approximately 11 s, which corresponds to the latent period between the Ca^2+^ signals generated by the CF and CW [32,33]. Thus, the latent period in the electrical responses of fertilized eggs is also prolonged by actin drugs, such as cytochalasin B and D [79]. Hence, at fertilization, the coordinated rearrangement of the egg cortical actin cytoskeleton may play a central role in modulating membrane electrical properties, Ca^2+^ signaling, and sperm entry [34,37].

While it has been postulated that the Ca^2+^ influx during the latent period is crucial for the initiation of the CW [80], it has been suggested that the polymerization status of the F-actin bundles within the MVs of the egg is an essential determinant of Ca^2+^ influx because of the Ca^2+^ channels located in the MVs [38,81]. Disruption of the actin filaments with actin drugs also leads to the ‘spontaneous’ generation of Ca^2+^ influx and Ca^2+^ waves in starfish eggs [62]. Additionally, the Con A pretreatment altered the structure of the CGs and their association with the egg membrane (Figure 4). CGs are linked to the egg plasma membrane through subplasmalemmal actin filaments [35,82], and their disassembly plays a role in shaping the pattern of the Ca^2+^ wave [36,38]. The morphological and functional changes in the actin cytoskeleton at the surface of the eggs are crucial for modulating fertilization-related Ca^2+^ events in *P. lividus* eggs [34,38], as illustrated in this study using Con A.

Our results indicate that the fluorescence from the lectin bound to the membranes of denuded eggs after insemination does not show an increase in the number of binding sites following the exocytosis of CGs, suggesting that new membranes are not incorporated into the egg membrane, contrary to previous suggestions [54]. Under the given experimental conditions, we found that treating denuded eggs with Con A impairs CGs exocytosis and inhibits the elongation of MVs on the surface of fertilized eggs (Figure 6), in contrast to denuded eggs without Con A, which showed extension of MVs (Figure 5). Denuded eggs exposed to Con A also showed a statistically significant reduction in CF and CW amplitude, which may be related to the morphological changes in the MVs and to the absence of CGs involvement in shaping the Ca^2+^ signals during fertilization [38].

Fertilization can occur even when sperm interact with denuded eggs that have undergone significant changes in their MVs morphology (Figure 5). Therefore, it is important to understand whether the fertilizing sperm primarily fuses with the egg membrane at the tip of the microvilli to transmit fertilization signals, as suggested by previous ultrastructural analyses [19,76], or if this interaction takes place at other regions of the egg membrane (as illustrated in the graphical abstract [30]). While several signaling pathways have been proposed to explain the complex intracellular Ca^2+^ mobilization activated during sea urchin egg fertilization [83,84,85,86,87,88,89,90], further studies are needed to uncover the molecular events underlying sperm-induced Ca^2+^ signaling.

Finally, our results support the notion that lectins may inhibit cell migration by regulating the content and distribution of F-actin [91]. In addition to its role as a cell surface marker for both normal and malignant cells in culture, our findings with sea urchin eggs indicate that Con A binding to the egg surface can transmit signals to the intracellular compartment. This interaction has the potential to disrupt multiple cellular signaling pathways, including the regulation of the actin cytoskeleton in the egg cortex and Ca^2+^ signaling. These results support the notion that lectins could be developed as therapeutic tools for targeting specific cancer cells [92].

## Figures and Tables

**Table 1:** Number of sperm incorporated into the intact or denuded eggs inseminated after preincubation with Con A in various conditions.

Status of the Egg Extracellular Matrix	Con A Dose in the Media (μg/mL), 5 Min Preincubation	Washing Out Con A Before Insemination	Sperm Counts in Egg: Mean ± S.D. (Number of Eggs Examined)
Intact	0	no	1 ± 0 (n = 60)
	1	no	1 ± 0 (n = 60)
	20	no	1 ± 0 (n = 60)
	200	no	0.33 ± 0.23 * (n = 60)
	1000	no	0 ± 0 * (n = 60)
Intact	0	yes	1 ± 0 (n = 40)
	1	yes	1.02 ± 0.08 (n = 40)
	20	yes	1.02 ± 0.08 (n = 40)
	200	yes	0.32 ± 0.23 * (n = 40)
	1000	yes	0 ± 0 * (n = 40)
Denuded	0	no	2.55 ± 0.62 (n = 60)
	200	no	0.8 ± 0.2 * (n = 60)

* *p* < 0.01.

**Table 2:** Thickening of the subplasmalemmal actin cytoskeletal layer visualized by LifeAct-GFP (Mean ± SD, μm).

	Intact Eggs	Intact Eggs + Con A (200 μg/mL)
Before fertilization	0.86 ± 0.34 (n = 6)	0.88 ± 0.17 (n = 6)
5 min after fertilization	1.97 ± 0.21 (n = 6)	1.20 ± 0.48 (n = 6)
* Paired *t*-Test	*p* < 0.001	N.S.
	Denuded Eggs	Denuded Eggs + Con A (200 μg/mL)
Before fertilization	0.76 ± 0.22 (n = 9)	1.24 ± 0.20 (n = 8)
5 min after fertilization	1.16 ± 0.44	1.55 ± 0.19 (n = 8)
Paired *t*-Test	*p* < 0.05	*p* < 0.01

* Note: Comparisons made in the same eggs before and after fertilization.

## Data Availability

All the data presented in this article, including the Supplementary Figures and Videos, are freely accessible according to the policy of MDPI.

## Supplementary Materials

The following supporting information can be downloaded at https://www.mdpi.com/article/10.3390/cells14231867/s1. Figure S1: Fluorescent Concanavalin A (Con A) binding to the surface of intact and denuded eggs. Upper row: Alexa Fluor 633 Con A, visualized by confocal microscopy, reveals the extracellular matrix in the VL of intact eggs. In a non-confocal setting with a broader pinhole aperture, the same Alexa Fluor 633 Con A revealed the jelly coat (JC) of intact eggs, which has been elusive to lectin detection (image on the right). Lower row: In the denuded eggs with the conventional 1 AU pinhole confocal setting, Alexa Fluor 633 Con A exhibits low-affinity lectin binding to the egg membrane, which becomes evident when comparing the intact eggs and denuded eggs lacking VL. In the non-confocal setting, it is apparent that the JC is removed (see the image on the right). Figure S2: Binding of Concanavalin A conjugated with Alexa Fluor 633 Con A to the jelly coat and vitelline layer of intact *P. lividus* eggs pre-injected with the Ca^2+^ dye using a CCD camera. (A) Intact eggs. (B) Eggs from which the jelly coat (JC) and vitelline layer (arrow) had been removed. Note the absence of the JC and a lower signal of the egg surface due to the binding of the lectin to the egg plasma membrane. Figure S3: Parameters of the Ca^2+^ response in fertilized eggs of *P. lividus*: (A) The relative increase in fluorescence intensity compared to time 0. (B) The relative increase in fluorescence intensity compared to the preceding time point. A detailed description of the parameters is provided in the main text. Figure S4: The dose-dependent effect of Con A on the Ca^2+^ response during the fertilization of intact eggs. *P. lividus* eggs microinjected with the dyes for Ca^2+^ measurements were preincubated with various doses of Con A for 5 min before insemination. The relative fluorescence (RFU) of the Ca^2+^ signal was obtained from a time-lapse recording after sperm addition. The moment of the first Ca^2+^ signal was set as t = 0. (A) The control (intact, not treated) eggs fertilized in NSW at pH 8.1 exhibited two modes of Ca^2+^ responses: the cortical flash (CF) and the subsequent Ca^2+^ wave (CW). The time interval between the two events is called the ‘latent period.’ (B-E) When intact eggs are exposed to various concentrations of Con A, there is a significant prolongation of the latent period and an alteration of the patterns of the sperm-induced Ca^2+^ response, as shown in the histograms in Figure 11. Video S1: A time-lapse overlay showing the fertilization response of an intact egg of *P. lividus*, illustrating the simultaneous cortical reaction and Ca^2+^ signals. In this video, the binding of the fertilizing sperm upon its arrival at the egg surface was set as t = 0. Two seconds later, a simultaneous increase in Ca^2+^ is observed in the cortex under the entire egg surface (cortical flash). Approximately six seconds after the sperm fuses with the egg, a Ca^2+^ wave is initiated. The propagation of this Ca^2+^ wave triggers contractions in the egg, resulting in a dimple at the site where the wave originates. This contraction separates the vitelline layer from the egg membrane, and the separation spreads across the entire surface of the zygote. Ultimately, this process leads to full elevation of the fertilization envelope through the exocytosis of cortical granules. Video S2: features a time-lapse overlay of the fertilization response in an intact *P. lividus* egg, showing the cortical reaction and Ca^2+^ signals after Con A treatment (200 µg/mL). The exposure of unfertilized, intact eggs to the lectin significantly compromises the fertilization response without completely blocking it. Unlike the non-treated eggs, eggs pretreated with Con A display a consistent delay of about one minute in initiating the Ca^2+^ wave following the onset of the cortical Ca^2+^ release (cortical flash). This time lag markedly prolongs the latent period. Additionally, there is a noticeable delay in dimple formation due to the changes in the egg’s contractility, which prevents the elevation of the fertilization envelope across the entire surface of the zygote. For further details, refer to the corresponding relative fluorescence graph in Figure 12 (the brown bold curve) and the histograms in Figure 11, which illustrate the alterations in the parameters of the Ca^2+^ response pattern. Video S3: A time-lapse overlay acquisition of the fertilization response of a denuded *P. lividus* egg to visualize the cortical reaction and Ca^2+^ signals. The binding of the fertilizing sperm to a denuded egg triggers a cortical Ca^2+^ flash two seconds later, leading to the initiation of a Ca^2+^ wave at the sperm–egg fusion site with a latent period of about six seconds, which is not significantly different from that observed in intact eggs. Monitoring the morphological changes at fertilization revealed subtle differences between denuded and intact eggs (compare it with Video S1). According to the bright field images here, activation of the denuded eggs by sperm did not trigger the formation of the dimple at the site of sperm fusion after the propagation of the Ca^2+^ wave. In addition, the time-lapse overlay video also revealed the lack of separation of the VL from the egg surface, as it had been removed before insemination. By contrast, the intact eggs at fertilization exhibited the characteristic dimple after the Ca^2+^ wave swept away from the sperm fusion and entry site (Video S1). For the corresponding relative fluorescence graph, refer to the blue bold line in Figure 12 and the analysis of other parameters related to the pattern of the Ca^2+^ fertilization response in Section 3. Video S4: A time-lapse overlay acquisition of the fertilization response of a denuded *P. lividus* egg inseminated after Concanavalin A (200 µg/mL, 5 min) treatment. When exposed to the lectin, the fertilization response of a denuded egg shows no significant differences in the initiation of the Ca^2+^ wave (latent period) following the onset of the cortical Ca^2+^ flash. As for the untreated denuded eggs, the removal of the vitelline layer before insemination affects egg contraction, dimple formation, and the elevation of the fertilization envelope from the zygote surface. For further details, refer to the corresponding relative fluorescence graph in Figure 12 (purple bold line) and the analysis of other parameters related to the pattern of the fertilization Ca^2+^ response in Section 3.
