# Supplementary figures and images for: The Binding of Concanavalin A to the Surface of Intact and Denuded Sea Urchin Eggs Affects the Fertilization Process by Altering the Structural Dynamics of Actin Filaments
Source: Cells. 2025 Nov 26;14(23):1867. doi: 10.3390/cells14231867 (PMC12691429; doi:10.3390/cells14231867)

Confocal

Intact

Non-confocal

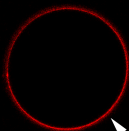

VL

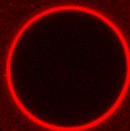

JC

Confocal

Denuded

Non-confocal

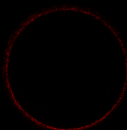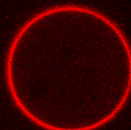

100  $\mu$ m

Supplement: Supplementary file 1 [file cells-14-01867-s001.zip › cells-3702569_Fig. S1.pdf]

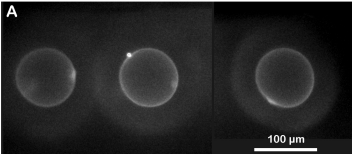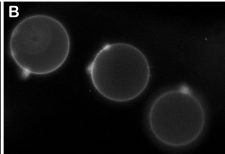

Supplement: Supplementary file 1 [file cells-14-01867-s001.zip › cells-3702569_Fig. S2.pdf]

**A**

$$RFU = (I_n - I_0) / I_0$$

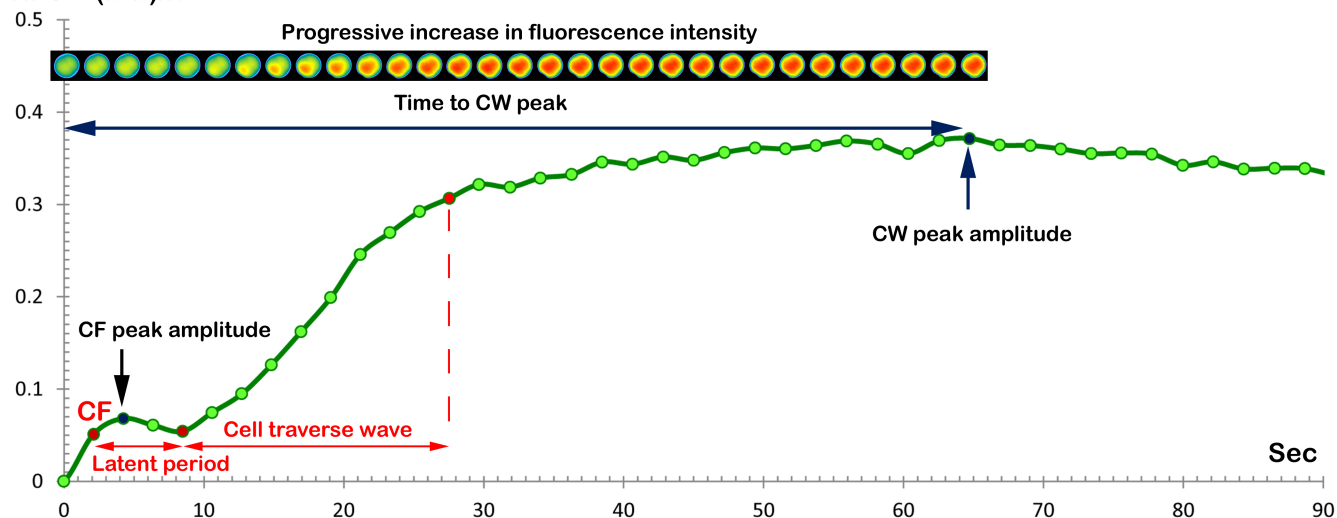

**B**

$$RFU = ((I_n - I_{n-1}) / I_{n-1}) \times 1000$$

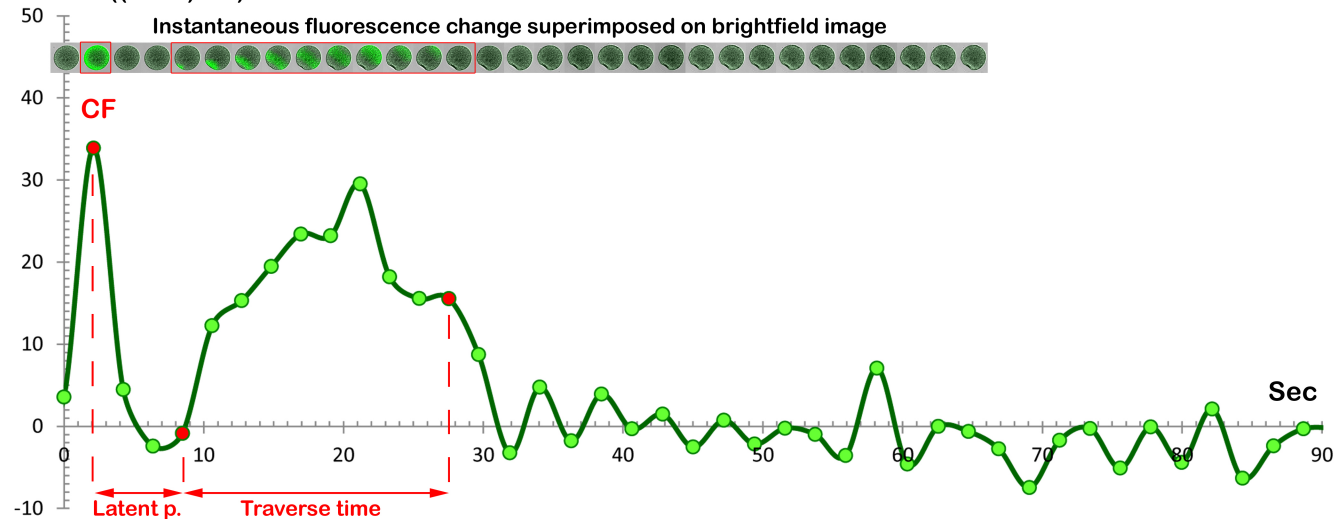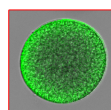

CF

Latent period

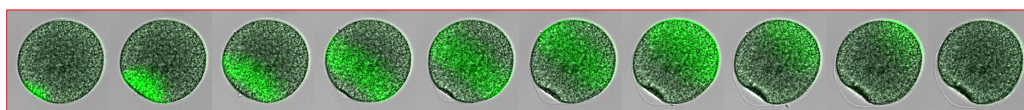

Traverse time

Supplement: Supplementary file 1 [file cells-14-01867-s001.zip › cells-3702569_Fig. S3.pdf]

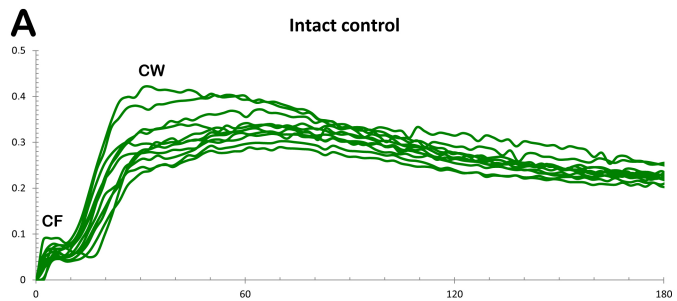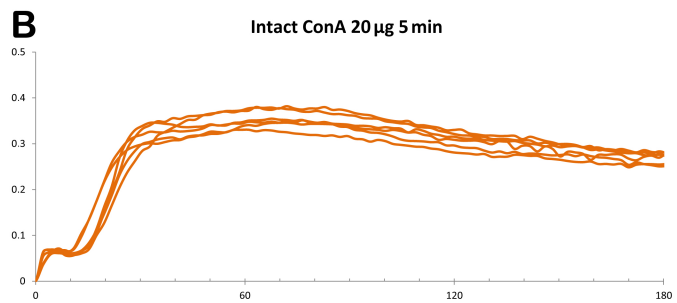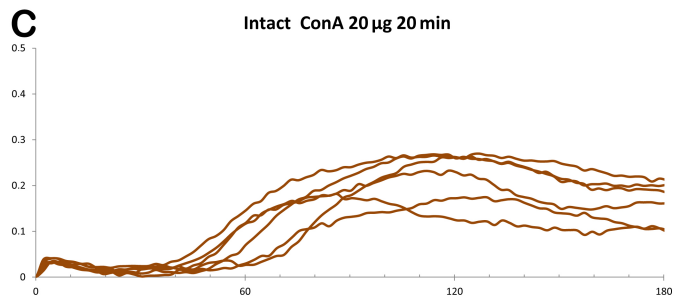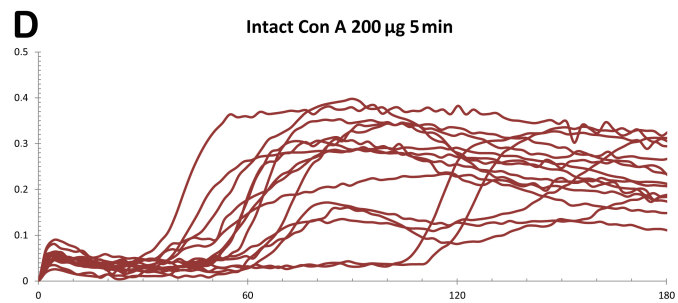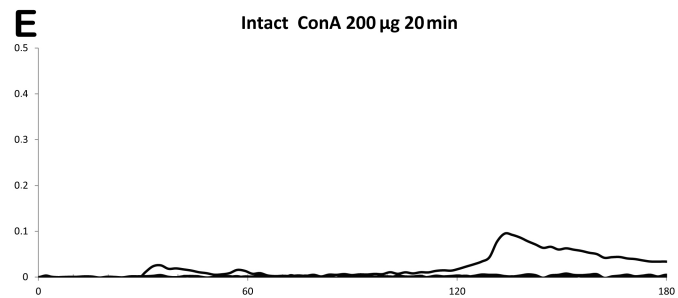

Supplement: Supplementary file 1 [file cells-14-01867-s001.zip › cells-3702569_Fig. S4.pdf]
